# Preparation for Medical and Dental Study

**Published:** 1880-10

**Authors:** 


### PREPARATION FOR MEDICAL AND DENTAL STUDY.

The following deliverance by Prof. J. E. Garretson, of the Philadelphia Dental College, given at one of his clinics, and recorded in the Dental Cosmos of August, is so true and well expressed that I cannot refrain from transferring it to the pages of the *Register.*

He evidently sees the evil referred to in all its deformity. It is the bane of the dental profession that any and every ignoramus who conceives that dentistry is a profitable business and easily learned, is permitted, and generally encouraged, to enter upon a special pupilage, preparatory to becoming an honorable member (?) of the dental profession.

Every member of any reasonable intelligence knows that the statements made by Prof. G. are correct.

There is not a dental professor or teacher in this country that does not know they are true, and yet the rule with our colleges has been and is yet in a great measure, to admit any and every one who applies with money enough to purchase tickets.

Oh for a deliverance for our profession. May it not be stabbed in the home of its friends. May it not be cursed in the efforts that are made to bless.

Prof. G. says:

"I hold in my hands a number of letters. Whence they come and from whom it is neither proper nor important that you should know. They furnish the text for my thoughts. Every one of these letters contains a word prominently projected,--diploma. In one other respect they are all alike,—showing incorrect spelling.

As your dean, gentlemen, I may be pardoned for intruding a little fatherly advice, for which the intention must be the excuse. It will prove a not unprofitable diversion of a few moments if I succeed in duplicating a lesson on the subject of incompatibles. What, for instance, if I ask you to consider as an example, the relation of a good diploma and bad spelling. Could a mixture of these be made, think you, which would deceive an intelligent public? Or, where would be the affinity between a parchment certifying proficiency and a tongue proclaiming deficiency? To carry dirt under one's nails is to bear about with him the earth of his grave. To spell incorrectly is to damn one's self. Do I never spell incorrectly? Don't inquire too closely. I do a hundred things in a way that permits of a better; constantly do, and have done, so many as to have forced into me the grace of charity. Happily, or unhappily, as the case may be, I speak to you out of experience.

Let me tell you the story of a first experience. Hopping out from the nest of a village office a long time before his pin-feathers were grown, a young man started once on the diploma business,—that is, he permitted himself to be called doctor; the right of the matter to the public and the wrong to himself he did not stop to consider; a dentist was a doctor and he was a kind of dentist. One evening shortly after this graduation a learned gentleman invited the doctor to spend an hour in his library. Soon the subject of an accident that had occurred during the day to a man's knee-pan came up; the savant appealed to the doctor for the technical name; the doctor scratched his head but failed to remember it. That, however, passed fairly well. The savant wanted next to talk about eyes; he wanted the name of the retinal ganglion. Myopia prevented the doctor seeing it. The savant had the audacity to ask what was taught at the college that made the man a doctor.

A diploma, even a faculty-manufactured diploma, is a satisfaction to a man only in proportion as he carries the meaning of it in his head. Nobody knows that better than the doctor who addresses you. He now has half a dozen, yet never for double a half-dozen years has he taken one of these out of its case, being ashamed to face it.

Some of you, young gentlemen, have written letters about diplomas. I hope the word has by this time almost gone from your minds; that you have become more intent on getting knowledge than parchments. I trust you have already found something very much better than a diploma, namely, the key to study. Find that, and a man has the "open sesame."

In our beautiful park your eyes not infrequently meet with signs bearing the inscription: "These flowers are under the care of the public." Knowledge is under the care of a man's self. Teachers teach, students learn, the mouth frames speech, but it is the ear that takes in the word. As dean, as professor, I have opposed and am opposed to compulsory professional education. Compulsion will no more make a scholar than will skill weave a sow's ear into a silk purse, or than will voice get information into ears having no sense of hearing or defiantly closed to instruction. Put the thing before the student, that is all; let him take or leave. The place does not exist where the opportunities to get information are greater than here in this great city of hospitals and general culture; the place is nowhere that less may be gained. It is for you, young gentlemen, to take or leave.

It is common for the inexperienced to prate of examinations as correctors of deficiencies. Examinations are bosh. A man with a good memory makes the professor's vote ten, while his mate with a poor one wins only five. I have been too long a teacher to be caught by any such chaff. I have heard a parrot pronounce well, but I have never seen the bird which could parse: I prefer a parser.

We are here learning to parse, not exercising memory. An item of principle is worth a note-book full of recipes. Get a principle and you have the recipes without the trouble of writing them down. I beg that you will lose no moment in studying unnecessary detail. Education has the meaning of an index, and the meaning of an index is to tell where to find a place or thing.

Apropos of an index, I own a dictionary, and my poor memory makes it necessary for me to consult it every day. It is by education that I know the meaning of a dictionary. Now, the writers of these letters held in my hand have, without doubt, poor memories. Am I to conclude them as well without education? Not necessarily: they may simply be careless. One or the other, however, assuredly they are. Either is alike inexcusable in a professional man. Don't forget the hint when you have letters to write. Faulty spelling is to be condoned only by a dean living in a house too full of windows to permit the throwing of stones.

A word more about the indices. In an index lies the whole meaning of college-life. You are to learn here of the things that pertain to professional education. You are not, however, expected to shovel these things into your brain. Books are repositories into which they are already shoveled. No man out of ten thousand has a memory that can get along without books. But what is the use of a gold mine if a man knows nothing of its location? Half the books written in English are Greek to English readers. The best investment a professional man makes of the earnings of his first few years of practice is in books, and in that education which teaches the use of books. Books tell a man all that is known,—about all that has been found out. A

man's most profitable companions are the volumes of his library. Buy books, young gentlemen.

Do not fret yourself with memorizing names. That is not at all the way to study. For example, let anatomy be considered. Who ever found himself able to do anything with it until he had exposed to him on the dissecting-table the secrets of the pages as expressed in our mnemonical associations? I think just now of the poor fellows, scattered all over the country, who are preparing to enter colleges next winter, and who are confounding their senses by the big words and long-drawn sentences of this heavy subject. You smile; and naturally. With the cadaver before them, they will behold a new revelation. The high wall will be seen to be nothing but a low fence.

A word about oral surgery, which seems to your speaker to have a marked future before it. This for the reason that a growing general intelligence expects and demands the assumption, on the part of somebody, of such duties as are implied in oral surgery; the somebody must know dentistry for the reason that oral surgery is to be understood alone by him who understands this art.

There is another reason why a near future must alter the status of the dental profession,—the saving of teeth is becoming less and less a mechanical act. Science is considering cause. To make exquisite fillings in teeth will for a very little time longer distinguish the dentist of the office from the metal-worker in a shop.

Still another reason is there,—education. We do what we know how to do. Many who know how to make fillings of all kinds have yet learned how to do more with prescriptions than can be done with the metals of the universe.

With brains replacing fingers, requirements are more quickly accomplished, and other tasks are undertaken; so, naturally, will all diseases of the mouth, jaws, face, teeth, and associate parts get under the care of one and the same practitioner; so, naturally, will there stand before the community specialists distinguished as oral surgeons.

